# Engraftment of Human Glioblastoma Cells in Immunocompetent Rats through Acquired Immunosuppression

**DOI:** 10.1371/journal.pone.0136089

**Published:** 2015-08-20

**Authors:** Peter C. Huszthy, Per Ø. Sakariassen, Heidi Espedal, Karl A. Brokstad, Rolf Bjerkvig, Hrvoje Miletic

**Affiliations:** 1 K.G. Jebsen Brain Tumour Research Centre, Department of Biomedicine, University of Bergen, Bergen, Norway; 2 Centre for Immune Regulation, Department of Immunology, University of Oslo/the National Hospital, Oslo, Norway; 3 Department of Clinical Science, University of Bergen, Bergen, Norway; 4 NorLux Neuro-Oncology Laboratory, CRP Santè, Luxembourg, Luxembourg; 5 Department of Pathology, Haukeland University Hospital, Bergen, Norway; Institute of Immunology, Rikshospitalet, NORWAY

## Abstract

Transplantation of glioblastoma patient biopsy spheroids to the brain of T cell-compromised Rowett (nude) rats has been established as a representative animal model for human GBMs, with a tumor take rate close to 100%. In immunocompetent littermates however, primary human GBM tissue is invariably rejected. Here we show that after repeated passaging cycles in nude rats, human GBM spheroids are enabled to grow in the brain of immunocompetent rats. In case of engraftment, xenografts in immunocompetent rats grow progressively and host leukocytes fail to enter the tumor bed, similar to what is seen in nude animals. In contrast, rejection is associated with massive infiltration of the tumor bed by leukocytes, predominantly ED1+ microglia/macrophages, CD4+ T helper cells and CD8+ effector cells, and correlates with elevated serum levels of pro-inflammatory cytokines IL-1β, IL-18 and TNF-α. We observed that in nude rat brains, an adaptation to the host occurs after several *in vivo* passaging cycles, characterized by striking attenuation of microglial infiltration. Furthermore, tumor-derived chemokines that promote leukocyte migration and their entry into the CNS such as CXCL-10 and CXCL-12 are down-regulated, and the levels of TGF-β2 increase. We propose that through serial *in vivo* passaging in nude rats, human GBM cells learn to avoid and or/ suppress host immunity. Such adapted GBM cells are in turn able to engraft in immunocompetent rats without signs of an inflammatory response.

## Introduction

When evaluating therapeutic approaches to be implemented in clinical oncology, using animal models with high relevance to human tumors is essential. We have previously established and characterized a patient biopsy xenograft model of glioblastoma multiforme in T cell-compromised nude rats, which has been applied in several studies of basic- and translational neuro-oncology [1–7], reviewed in [8]. In this model, the tumor tissue is mechanically dissociated and adapted to agar-overlay cultures to allow the formation of spheroids between each *in vivo* passaging stage. A considerable advantage of the spheroid model compared to cell line-based models is preservation of the patient genotype [5, 9]. In particular, *EGFR* amplification, a hallmark genetic aberration within GBMs is frequently lost/selected against in standard monolayer serum culture, but preserved in biopsy spheroids and in xenografts [6, 10]. In general, lack of communication between human and rat antigens and the immune-compromised nature of the host diminish the translational relevance of results obtained from xenograft tumors. On the other hand, syngeneic rodent models where the host harbours a complete immune system are based on genetically and phenotypically homogenous cell lines, which poorly resemble the heterogeneous tumors found in humans. Tumor cells in syngeneic models generally fail to show diffuse infiltration into the host brain, which is a prominent hallmark of human GBMs. Therefore, the establishment of an infiltrative GBM model based on human xenograft material growing in immunocompetent animals would be desirable. Although human GBM tissue has previously been transplanted to the anterior eye chamber and the brain choroidal fissure of immunocompetent rodents [11–13], a reliable model for human brain tumors has not been established due to low engraftment rates. Furthermore, the mechanisms that govern GBM xenograft tolerance in rodents have not been well characterized; most of our knowledge relating to tissue engraftment in the rat CNS derives from transplantation experiments aimed at correcting neurodegenerative disorders [14].

Here, we assessed xenograft engraftment rates, host survival, dominant leukocyte populations and cytokine responses in an effort to establish an animal model for human GBMs in immunocompetent Rowett rats. We show that human GBM tissue serially passaged in nude rat brains may engraft in immunocompetent littermates in contrast to spheroids made directly from patient biopsies. We investigated some possible adaptation mechanisms that may have facilitated the tolerance of human tumor xenografts in fully immunocompetent rats.

## Material and Methods

### Ethics statement

Primary GBM biopsies were obtained at the Department of Neurosurgery, Haukeland University Hospital, Bergen. All patients gave a written informed consent for tumor biopsy collection and signed a declaration permitting the use of their biopsy specimens for research. The study was approved by the Norwegian Regional Research Ethics Committee (Rek-Vest, approval number 013.09). All animal protocols were approved by authorities in an AAALAC-accredited animal facility at the Haukeland University Hospital and were in accordance with the national regulations of Norway. Case approval numbers were 08/38978-2008120 and 08/110915-2008350.

### Spheroid culture

Spheroid cultures were established as previously described [15]. Briefly, tissue samples were minced into 0.5-mm fragments and placed into 80-cm^2^ tissue culture flasks (Nunc, Roskilde, Denmark) base-coated with 0.75% agar (Difco, BD Biosciences, Franklin Lakes, NJ). The spheroids were maintained in standard eukaryotic cell culture conditions.

### Animals

Rowett nude rats were maintained in our facility by breeding homozygous males (*rnu/rnu*) with heterozygous females (*rnu/-*); the latter have a normal T cell physiology [16]. Homozygous and heterozygous mutants are easily distinguished by observing body hair growth. Rats of both sexes, between 8 and 12 weeks of age were used. Animals were sacrificed by CO_2_ inhalation after observing radiological signs of excessive tumor burden or in cases of rejection. In other cases, animals were taken after observing neurological symptoms of intracerebral tumor burden.

### Spheroid implantation

Implantation of tumor spheroids were performed as described [3]. Sedation and anesthesia was induced using a combination of Domitor (medetomidine, 0.4 mg/kg) and Ketalar (ketamine, 60 mg/kg). Local anaesthesia, Marcaine, was given under the scalp (bupivacaine, 2.5 mg/ml inj., 1 ml given) before incision. Thereafter, a burr hole was drilled 1 mm posterior to the bregma and 3 mm to the right of the midline suture, and 15 spheroids (diameter, 400μm) were implanted into the cerebral cortex at 2.5 mm depth using a Hamilton type 7125 syringe (Hamilton, Bonaduz, Switzerland). The skin flaps were closed with suture and the rats were returned to their cages after 30 minutes of rest in baby incubator set at 32°C. The analgesia protocol also included s.c. application of Temgesic (buprenorphine, 0.05 mg/kg) for postoperative pain care if required.

### Magnetic resonance imaging

Tumor growth was visualized using a Bruker Pharmascan 7 Tesla MR scanner (Bruker Biopsin MRI GmbH, Ettlingen, Germany) using T2-, and occasionally T1 (with gadolinium contrast agent) sequences as previously described [2].

### Evaluation of xenograft engraftment

Assessment of engraftment was done by evaluating MR images and histology. The animals were divided into two groups:

1. tumor rejection/immune system activation: no engraftment or the evidence of rejection on MRI, and proof of rejection on histology (various-sized lesions with significant leukocytic infiltration into the tumor bed concomitant with the presence of perivascular leukocytes in the normal brain).
2. tumor engraftment/tolerance: progressive xenograft growth on MRI (comparable to nude rats), full-sized tumors at sacrifice without leukocyte infiltration into the tumor bed, no perivascular leukocytes in the normal brain tissue.

### Fluorescent double staining on frozen tissue sections

Fluorescent double staining was performed using combinations of human specific rabbit anti-nestin (AB5922, Chemicon, 1:200), rat-specific CD45 (#554875, BD, 1:200) antibodies and appropriate secondary reagents.

### Immunohistochemistry on frozen tissue sections

Immunostaining against rat-specific CD markers was performed on frozen brain sections from saline-perfused animals. After fixation and blocking, the sections were incubated for 1 hour with mouse anti-rat antibodies (all purchased from BD Pharmingen, Franklin Lakes, NJ), used at the following dilutions: CD4 (554841, OX-38, 1:100), CD8a (554854, 1:600), CD45 (554875, OX-1, 1:500), CD161a (NKR-P1A, 555006, 1:400), Dendritic Cell Antigen (555010, OX-62, related to CD103), 1:400; CD68 (ED1, 550305, 1:100). The sections were developed using horse-anti mouse biotinylated secondary antibodies and subsequently avidin-biotin complexes, using 3–3`-diaminobenzidine as substrate.

### Immunostaining on paraffin-embedded tissue sections

Dilutions of primary antibodies were: anti-Granzyme B (Abcam, Ab4059), 1:75; anti-Foxp3 (Biolegend, clone 150D), 1:50; anti- AIF-1 (LifeSpan Biosciences, Nordic Biosite, Oslo, Norway), 1:100, anti-Cathepsin S (sc-6503, Santa Cruz Biotechnology, Heidelberg, Germany), 1:100. The sections were developed using the DAKO Envision system with diamidobenzidine as substrate.

### Quantification of immunostained antigens

After capturing eight to ten x400 fields per case for each of the antigens, the images were saved on a Nikon light microscope and associated software (Nikon, Tokyo, Japan). The numbers of positive cells or the area fraction occupied by positive cells per high power field were counted.

### Blood collection for cytokine analysis

Blood was harvested through the saphenous vein from twelve immunocompetent rats implanted with P3 high generation spheroids. The blood was collected into heparinized Microvette tubes (Sarstedt, Numbrecht, Germany). Blood samples were centrifuged at 1000G for 10 minutes. Isolated sera were stored at -80°C until analysis, performed within 4 months from the day of storage. Animals were followed by biweekly MRIs. Rats with large tumors were followed closely and sacrificed at the earliest onset of symptoms, whereas symptom-free rats were taken on day 90 p.i. Tumor histology was assessed by microscopy.

### Quantification of serum cytokine levels

Serum samples were run on a fluorescence-based rat-specific cytokine 11-plex for quantification (Millipore, Oslo, Norway). The following cytokines were included: GM-CSF, IL-1α, IL-1β, IL-2, IL-4, IL-6, IL-10, IL-17, IL-18, IFN-γ and TNF-α. Serum samples were taken on days 0, 4, 7, 11, 18, 43, and 53 p.i.

### Quantitative RT-PCR

Brain sections were cut in a cryotome under sterile conditions for RNA isolation. The first and last section was stained with haematoxylin and eosin to confirm the presence of tumor in the sections that were analysed. Human-specific primers were designed using the Primer Blast Program (NCBI). Lack of cross-hybridization with the rat-specific form was confirmed with Primer Blast and run on cross-reaction control samples. For assessment of human transcripts, the human Cytokines & Chemokines RT² Profiler PCR Array representing 84 key molecules (Qiagen, Oslo, Norway) was run on samples from xenografts of the same original patient biopsy.

### Statistical analysis

Groups were analyzed for normal distribution by the Shapiro-Wilk Test significance (SPSS). For immunopositive cell counts and comparison of cytokine concentration values, groups were compared using the Mann–Whitney *U*-test, where two-tailed exact significance values are reported. Several groups were compared using the Kruskal-Wallis test. For comparisons of engraftment status and the presence of intracerebral immune response between animal groups, the Fischer`s Exact Test was utilized and two-sided cumulative P values (sum of small P´s) were reported.

## Results

### Human glioblastoma xenograft engraftment in immunocompetent rats

**Fig 1:**
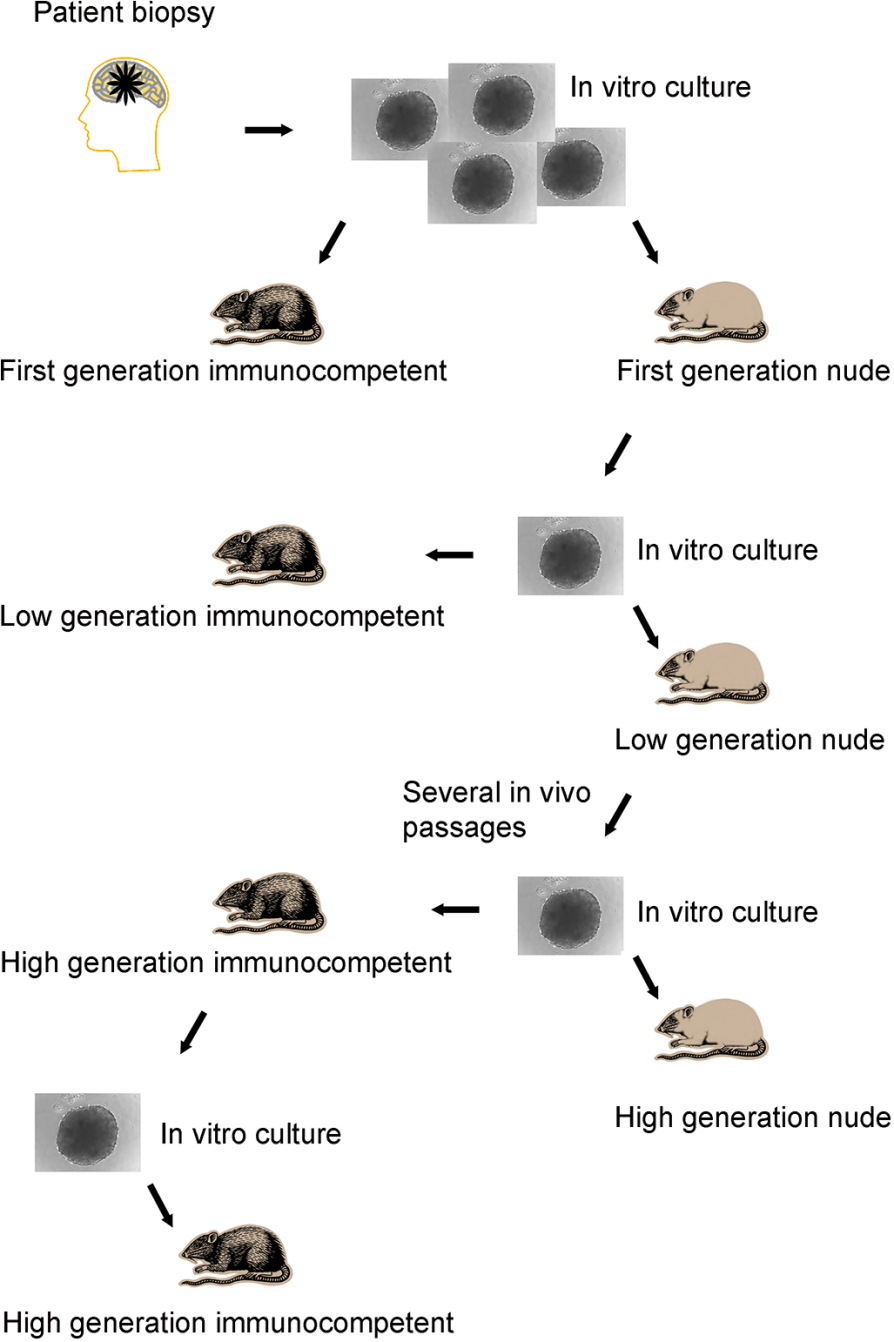
Outline of transfer experiments performed in nude and immunocompetent rats. Biopsy tissue from GBM patients or passaged xenograft tumors were minced into cubes and allowed to form spheroids in agar-overlay cultures before transplanting to the brain of animals. Comparisons in engraftment were made between 1) immunocompromised nude versus immunocompetent animals implanted with spheroids from the same culture; both primary, low generation and high generation material, 2) primary/low generation versus high generation spheroids in immunocompetent animals, 3) xenografts generated from tumors that engrafted in nude versus immunocompetent animals concerning subsequent engraftment rate in immunocompetent animals.

**Fig 2:**
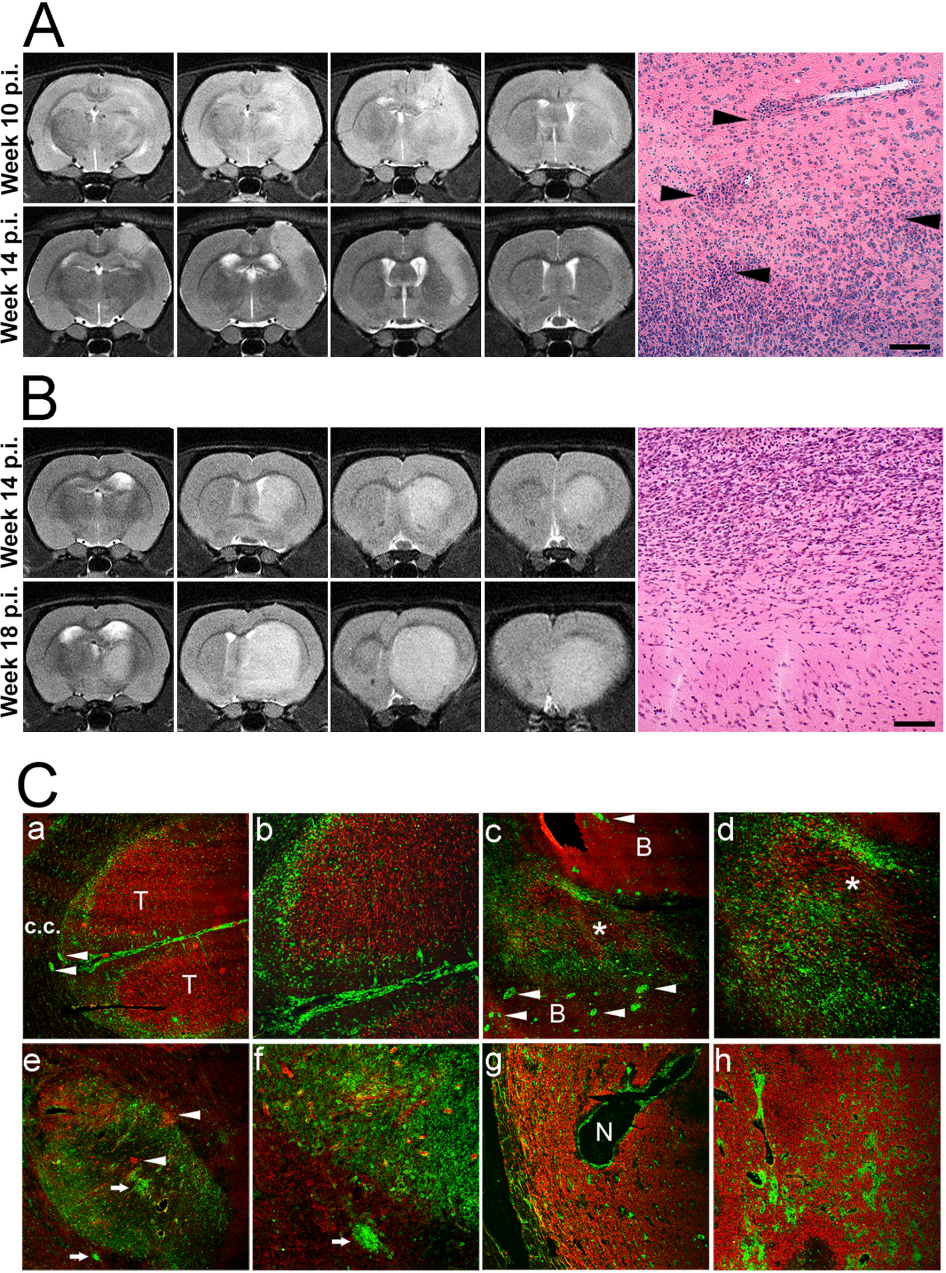
GBM xenograft rejection in immunocompetent rats evidenced by MRI and immunohistology. (A, top row) Serial MRI sections (from left to right) show a lesion that appeared ten weeks p.i. of low generation spheroids. The xenograft presented with a diffuse, weakly hyperintense signal on T2-weighted images without a clear demarcation toward the brain parenchyma (upper panel). Four weeks later, there is a reduction in the volume of the hyperintense area, and the lesion now shows a demarcated border toward the brain. Micrograph: Arrowheads point to perivascular (top) and peritumoral (bottom) leukocytic infiltrates in the brain. (B) Serial MRI slices representative of progressive tumor growth. The upper panel shows slices of the lesion fourteen weeks post implantation. The lower panel shows expansion of the tumor four weeks later. Control injections with PBS only did not produce any MRI signal apart from an outline of the needle track (hypo-intense) on early scans. Micrograph: No infiltration of leukocytes in the brain or around the tumor. Scale bars: 50 μm. C. Immunofluorescent micrographs show xenograft rejection (a to f) and tolerance (g, h). GBM cells are marked by human-specific nestin (red) and host cells by rat leukocytic common antigen (CD45, green). (a,b) Early phase of rejection. The tumor bed (T) is surrounded by a band of host leukocytes. CD45+ cells are observed in the meninges and perivascularly (arrowheads) in the brain. c.c.: corpus callosum. (c,d) Later stage of rejection. The tumor (asterisk) is infiltrated by host leukocytes. In the surrounding brain (B), numerous microvessels have perivascular cuffs indicating recruitment of leukocytes from the circulation (arrowheads). (e,f) Complete rejection. In the tumor bed, only islands of tumor cell foci remain (arrowheads). (g,h) Tolerance. A full-sized, vascularized tumor. Leukocytes are mainly seen around necrotic areas (N) and around tumor blood vessels. Infiltration into the tumor bed is limited. Original magnification of the figures; a, e, c, g: x50; b, d, h: x100, f: x200.

We transplanted biopsy spheroids derived from six patients diagnosed with primary GBM to the right hemisphere of immunocompetent and nude rats. Three specimens were generated directly from patient biopsies (primary spheroids), one specimen was passaged once in a nude rat (low generation) and two specimens were high generation spheroids that have undergone multiple transplantation cycles in nude rats (for an overview, see Fig 1). High generation xenografts P3 and P8 (denoting Patient tumor 3 and 8) have been characterized and published [1, 6]. We assessed initial engraftment using T2-weighted MRI. The xenografts appeared as weakly hyperintense, diffuse lesions discernible from three weeks post-implantation. Visible lesions appeared in all nude rats and grew progressively. On the other hand, initial lesions appeared in only 20 of 45 (44%) of immunocompetent animals, when evaluated starting three weeks p.i. We followed the animals by bi-weekly MRIs and noted that all primary- and low generation xenografts were rejected, whereas one out of three high generation tumors engrafted successfully (*P* = 0.016; Table 1). Rejection was evident when the slightly hyperintense area associated with xenograft tissue gradually decreased on successive time points (Fig 2A). Progressive growth was associated with a successive increase of the tumor size (Fig 2B) until the animals reached the humane endpoint. Longitudinal follow-up suggested that xenograft rejection was complete, i.e. a subpopulation of cells did not escape and regrow, at least within the first five months. Histological analysis of xenografts that underwent rejection revealed accumulation of leukocytes at the tumor border and around brain blood vessels (Fig 2A). In case of tolerance, leukocytes did not infiltrate the xenografts or enter the normal brain tissue (Fig 2B).

**Table 1:** Engraftment outcome of GBM biopsy spheroids in immunocompetent rats.

	Primary/low generation xenografts (%)	High generation xenografts (%)	*P* value
Number of xenografts that appeared on initial MRI scans	6/21 (28.6)	14/24 (58)	0.0715
Number of xenografts rejected after appearing on MRI scans	6/6 (100)	6/14 (42.9)	**0.0419**
Number of xenografts that engrafted	0/21 (0)	8/24 (33.3)	**0.0160**

Next, we evaluated if one successful engraftment event in an immunocompetent rat brain led to complete adaptation to the immunocompetent host. We euthanized nude- and immunocompetent rats that developed full-blown brain tumors, established spheroids in short-term culture and implanted them into new immunocompetent recipients (Fig 1). We found that both P3 and P8 high generation spheroids taken from an immunocompetent animal engrafted at a 50% rate (S1 Fig), similar to what was seen for spheroids derived from nude rats (*P* = 0.96 for P8 and *P* = 0.71 for P3). This indicated that one successful engraftment event in an immunocompetent animal did not lead to complete adaptation to the immunocompetent host.

### Leukocyte infiltration of human GBM xenografts growing in immunocompetent rats

Co-staining of tumor cells (human-specific nestin) and leukocytes (rat-specific leukocyte common antigen or CD45) revealed the pattern of host cell infiltration in the xenografts (Fig 2C). Representative images taken at various stages of the rejection process (Fig 2C, a-f) are compared to a typical case of tolerance (Fig 2C, g-h). During early stages of rejection, the tumor tissue was typically surrounded by a ring of leukocytes. Infiltration of the meninges and perivascular areas of the brain was observed as well (2C, a-b). Subsequently, leukocytes colonized the graft and infiltrated the tumor bed (2C, c-d). Toward the end of the rejection process, only pockets of single glioma cells remained among foci of leukocytes (Fig 2C, e-f). Tolerance was characterized by the establishment of full-sized xenograft lesions, similar to those seen in nude rats. Leukocytes were present around tumor blood vessels, but the tumor bed was not infiltrated (2C, g-h). In the case of tolerance, the meninges and normal brain blood vessels were devoid of leukocytes.

### Assessment of leukocyte subsets involved in GBM xenograft rejection

**Fig 3:**
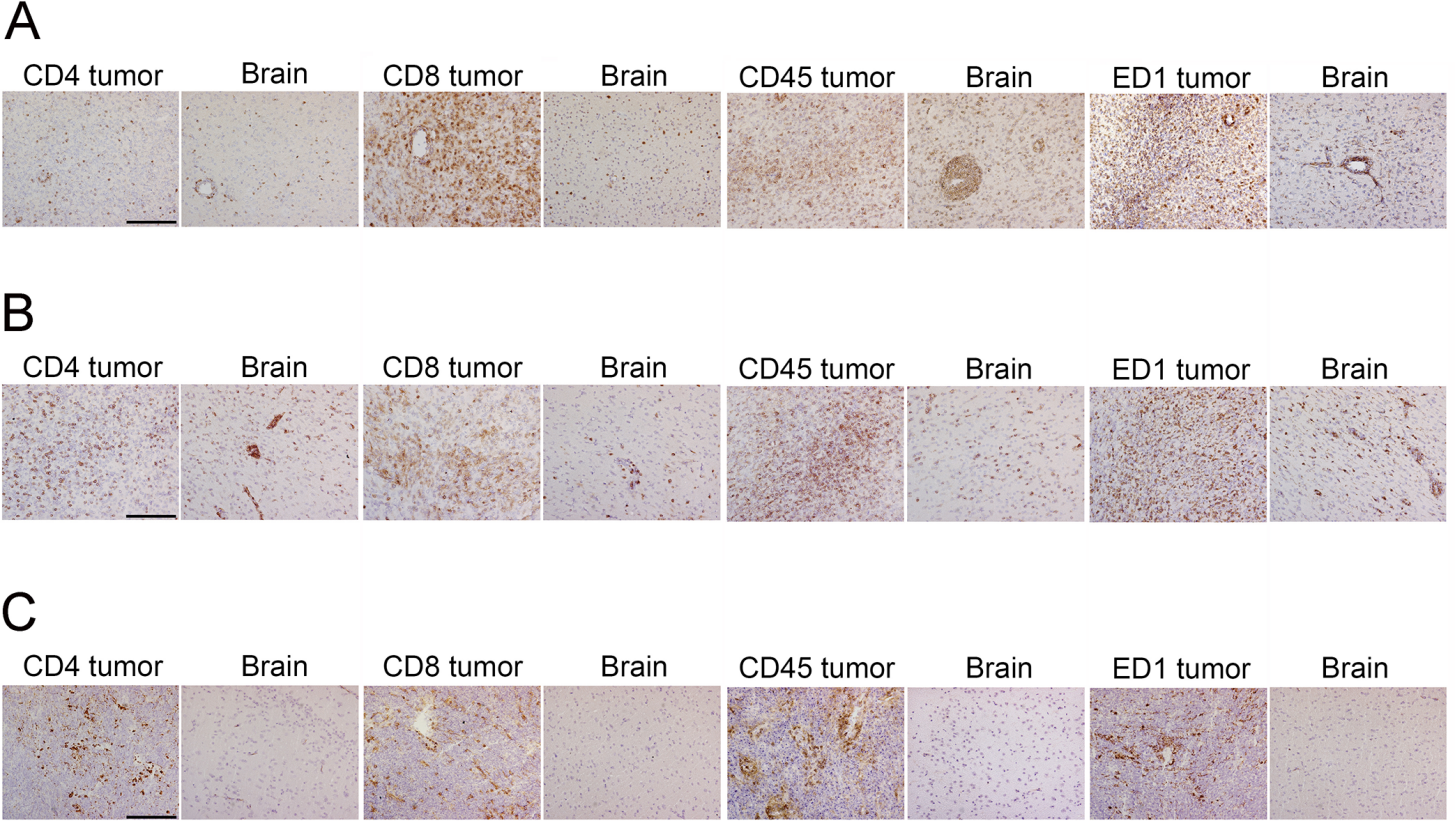
Monocytes and T cells infiltrate the tumor bed and the brain of immunocompetent rats that reject their xenografts. Panels show typical distribution of leukocyte subsets in the tumor and brain tissue of immunocompetent rats implanted with GBM xenografts. Brain slices were stained for the indicated markers and x400 fields were captured from representative areas. Shown are typical cases from immunocompetent rats with different engraftment outcomes. (A) An infiltrative GBM xenograft generated directly from patient tissue that elicited a strong immune response. (B) A diffusely growing, high generation GBM xenograft with significant immune response. (C) High-generation GBM xenograft, tolerance. Scale bar: 100 μm. ED1 antigen: CD68.

**Fig 4:**
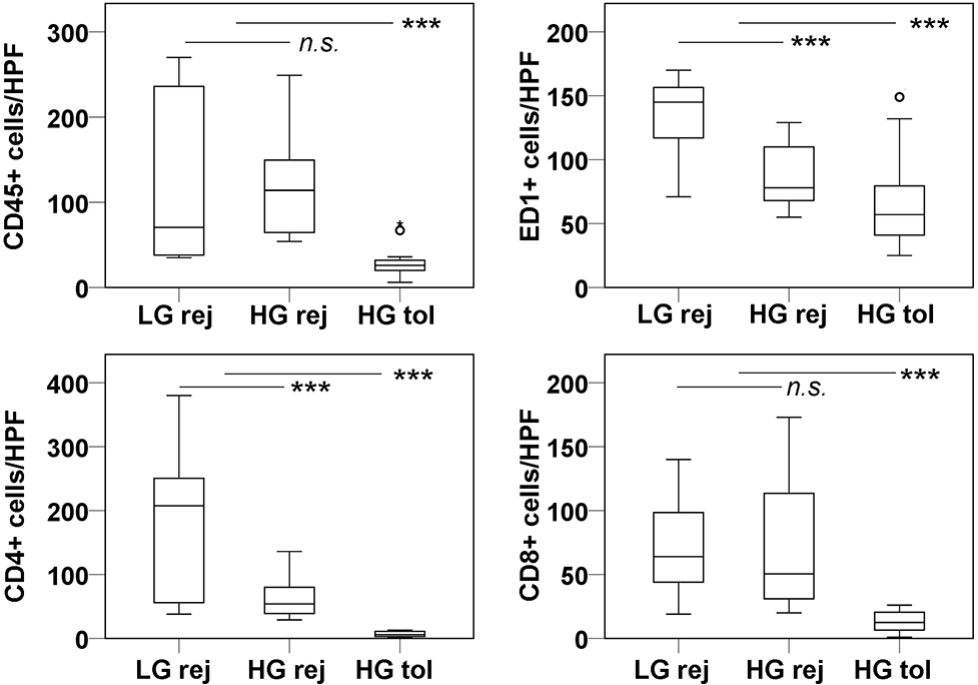
Leukocytes, therein T cells and monocytes are increased in the tumor bed in rats undergoing xenograft rejection. Box plots show the distribution of CD45+, CD4+, CD8a+ and ED1+ cells per high power field (HPF, x400) taken from immunostained sections. Three categories of tumors were considered, low generation xenografts undergoing rejection, high generation xenografts undergoing rejection and high generation xenografts with tolerance. The groups were compared using Mann-Whitney *U* Test. Significant differences are denoted by asterisks (*** marks *P*<0.001); n.s.: not significant. Circles represent outlier data points.

In the tumor bed of low- and high generation xenografts which underwent rejection (Fig 3A and 3B), ED1+ macrophages and microglia were numerous, together with CD4+ and CD8a+ lymphocytes. These cells were also found surrounding the xenografts, as well as in normal brain tissue. ED1+ cells predominantly adapted a spherical (presumably macrophages) or an amoeboid (activated microglial) morphology. NKR-P1A (CD161a)-expressing Natural Killer cells and cells expressing Dendritic cell antigen (DCA or OX-62) were less frequent in the xenografts, and very scarce in the brain (S2 Fig). In the case of tolerance, all leukocyte subsets were predominantly confined to perivascular areas within the tumors, and the brain tissue was not infiltrated (Fig 3C). Quantification revealed that the total numbers of leukocytes, and therein T cells and microglia/macrophages were significantly higher in xenografts with rejection versus tolerance (median values ± SD: CD45: 94 ± 74.6 vs. 26 ± 16.83, CD4: 60.5 ± 102.0 vs. 6.0 ± 4.1, CD8a: 59 ± 47.3 vs. 12.5 ± 8.30, ED1: 114 ± 35.0 vs. 57 ± 34.7 vs.; for all *P*<0.001; n = 6) (Fig 4A–4D). In tumors which underwent rejection, the numbers of CD4+ cells and ED1+ cells were higher in low generation tumors than in high generation tumors (207.5 ± 124.0 vs. 54.0 ± 34.9, *P* = 0.005; 145 ± 27.6 vs. 78 ± 25.5, *P*<0.001). The total number of leukocytes, and of CD8+ cells were not different between these groups (CD45: 70.5 ± 101.6 vs. 114 ± 53.8, *P* = 0.675; CD8a: 64 ± 35.0 vs. 50.5 ± 51.0, *P* = 0.521).

**Fig 5:**
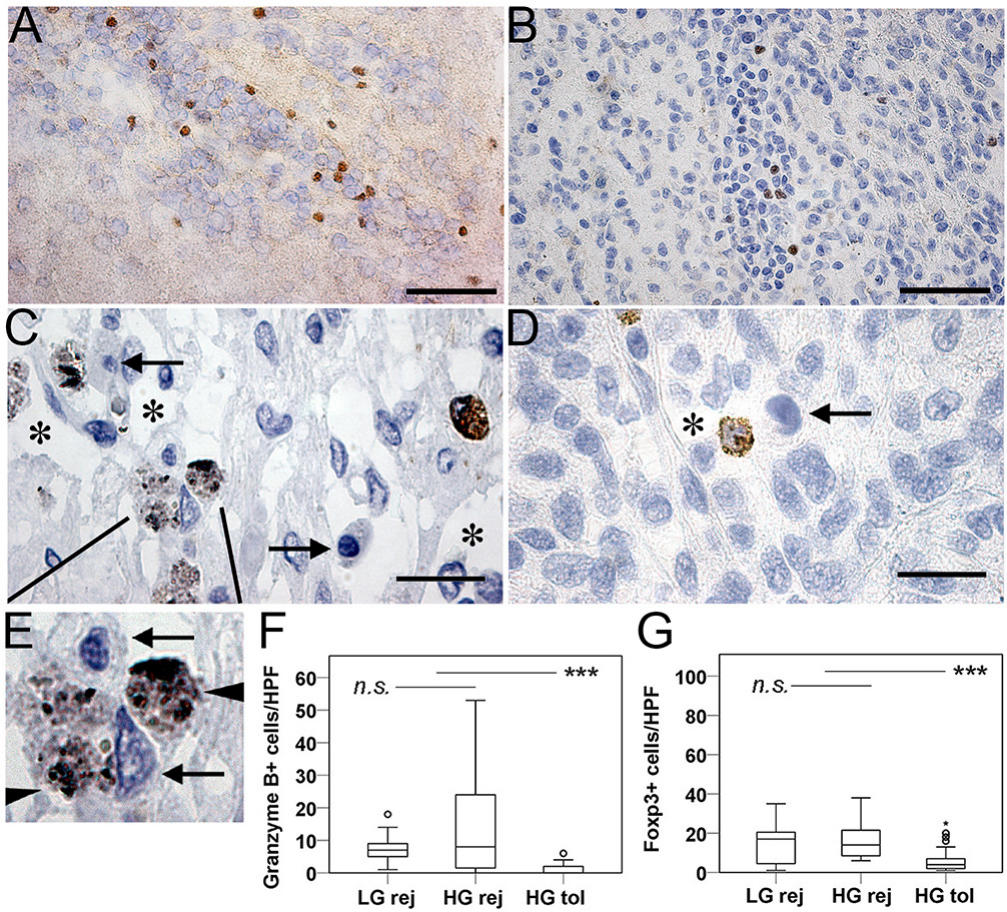
Cytotoxic effector cells and regulatory T cells in GBM xenografts growing in immunocompetent rats. (A, B) Representative images show Foxp3 immunopositive perivascular lymphocytes in a rejected (A) and a tolerated (B) xenograft. Foxp3+ cells were scarce, most often found in the vicinity of tumor blood vessels. Scale bars: 50 μm (C, D). Granzyme B-positive cytotoxic cells in a GBM xenograft with rejection. Asterisks mark tissue lyzed by effector cells. Magnified view (E) shows granzyme-containing lysosomes (arrowheads) and target cells with apoptotic nuclei (arrows). (D) Generally, tolerated tumors were devoid of cytotoxic cells, but some were found perivascularly. Scale bars: 20 μm. (F) Quantification of Granzyme B expressing cells. (G) Quantification of Foxp3 expressing cells. Significant differences are denoted by asterisks (*** marks *P*<0.001); n.s.: not significant. Circles represent outlier data points.

We assessed the contribution of regulatory T-cells by anti-Forkhead box P3 (Foxp3) immunostaining. Positive cells were found mostly around tumor blood vessels (Fig 5A and 5B), and sparsely disseminated in the xenograft tissue. There were more Foxp3+ cells in xenografts undergoing rejection versus tolerance (15.5 ± 15.64 vs. 4 ± 5.72 per field, *P*<0.001). However, when adjusting for the differences in total number of CD4+ T cells, the ratio of Foxp3+ cells to CD4+ T cells was found to be 2.6 times higher in tolerated versus rejected xenografts (a ratio of 0.67 vs. 0.26, respectively).

We assessed the contribution of cytotoxic effector cells by staining for Granzyme B, which identified pro-apoptotic granules in activated killer cells. In xenografts with on-going rejection, cytotoxic cells were in close contact with glioma cells in the tumor bed (Fig 5C). Granzyme B+ cells were very scarce in tolerated xenografts, and if found, these cells were in or around tumor microvessels (Fig 5D). Apoptotic nuclei, presumably from tumor cells, were evident in the vicinity of Granzyme B+ lymphocytes (Fig 5E). There were higher numbers of Granzyme B+ cells in tumors with on-going rejection versus tolerance (median values ± SD: 7 ± 11.31 vs. 0 ± 1.30; *P*<0.001, Fig 5F).

### Analysis of serum cytokine levels

**Fig 6:**
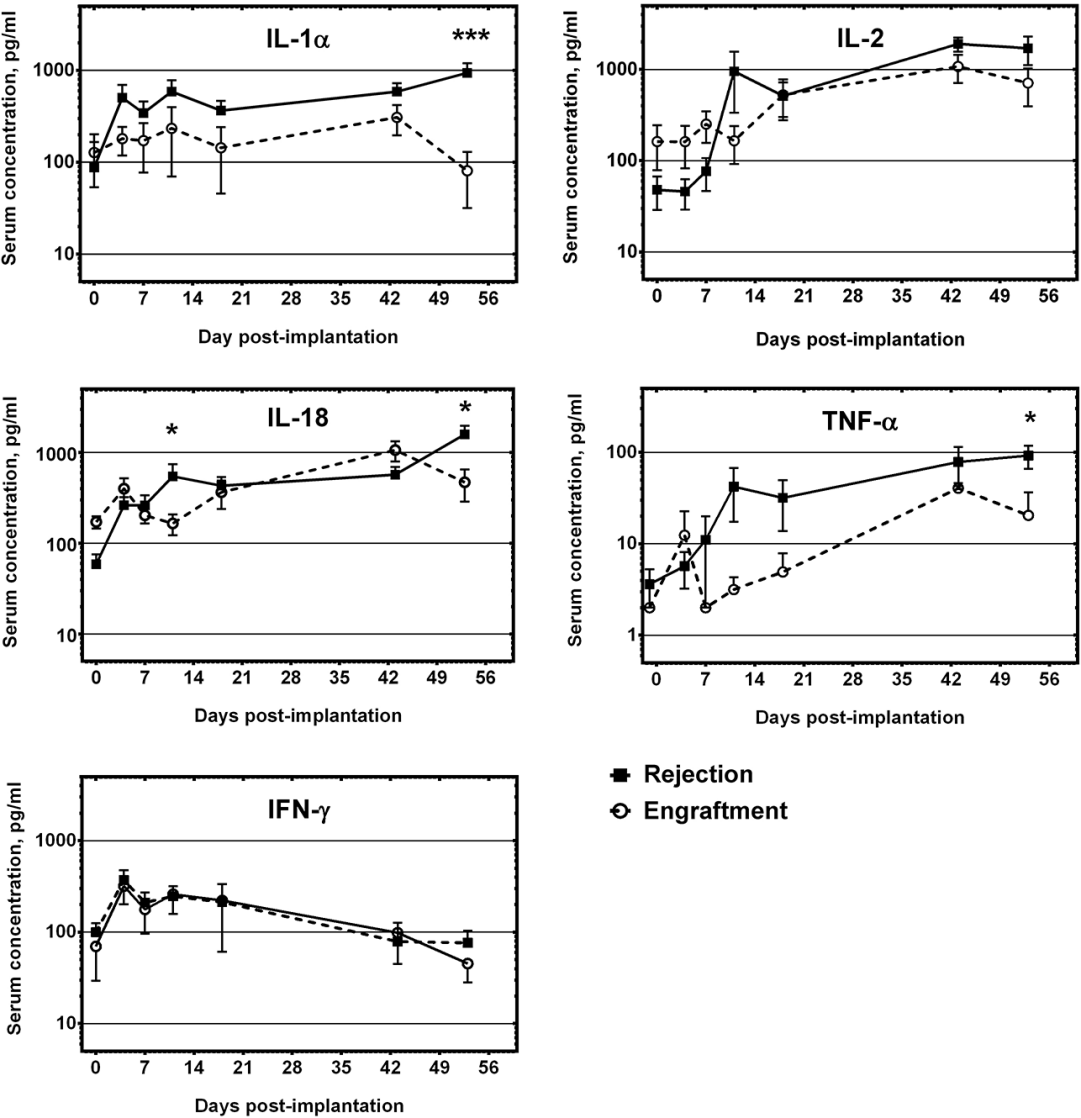
Serum levels of selected cytokines in immunocompetent rats implanted with GBM xenografts. Shown are the serum concentrations of cytokines that were consistently detected throughout the time course of the experiment. X axis indicates days from implantation (d = 0). The lines show average serum concentrations (± SEM) in the rejection group (black squares) vs. the engraftment group (open circles) of rats implanted with high generation P3 spheroids. Significant differences are denoted by asterisks (*** marks *P*<0.001, * marks *P*<0.05).

To assess the systemic responses to GBM xenografts growing in immunocompetent rats, we analysed sera from a group of animals (n = 12) implanted with high generation P3 spheroids. We compared serum cytokine concentration values for animals with radiological and histological proof of rejection versus tolerance. Of the panel of cytokines evaluated (see Material and Methods), IL-1α, IL-2, IL-18, TNF-α and IFN-γ were present in the sera of the majority of animals (Fig 6), whereas all the other cytokines were present only sporadically. The levels of IL-1α, IL-2, IL-18 and TNF-α generally increased throughout the time course of the rejection process, whereas the levels of gamma interferon declined. We observed peaks in cytokine levels at an early time point (11 days post-implantation) and later (43–53 days post-implantation). We found that the levels of IL-1α, IL-18 and of TNF-α were significantly higher in rats that rejected their tumors versus in rats with tolerance at given time-points (Fig 6), whereas IFN-γ levels did not correlate with rejection.

### Adaptation of human GBM cells to the xenogeneic host during passaging in nude rats

**Fig 7:**
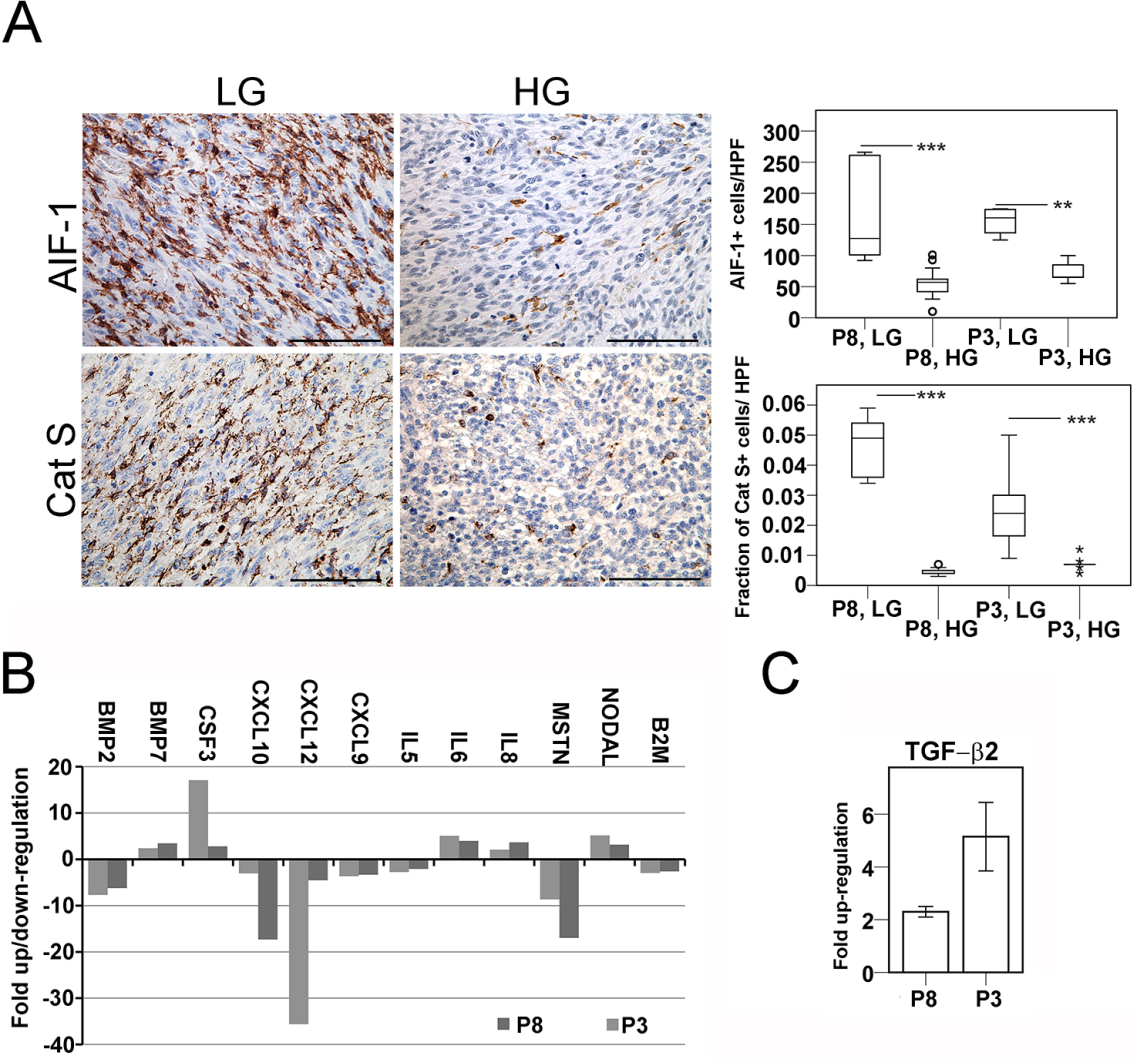
Repeated *in vivo* passaging cycles of human GBM xenografts in nude rats lead to loss of immunogenicity. A. Left: In low generation xenografts, Allograft Inflammatory Factor-1 and Cathepsin S staining identified tumor-infiltrating microglia with predominantly intermediate and amoeboid morphology. Right: In high generation xenografts, immunopositive cells were less abundant. Box plots compare the numbers of AIF-1+ cells, or the area fractions occupied by Cat S+ cells per high power field (HPF, x400). Significant differences are denoted by asterisks (*** marks *P*<0.001, ** marks *P*<0.005). Circles represent outlier data points. B. Fold change comparison of transcript levels of selected human cytokines, chemokines and growth factors with relevance to immune responses in low- versus high generation xenografts. Of the panel of analysed transcripts in the array, only those with at least a three-fold change in levels between low and high generation in both patient xenografts are presented. C. Fold change of tumor-derived TGF-β2 transcripts in corresponding low and high generation xenografts. Mean ± SEM; three independent runs. Scale bars in A: 100 μm.

An adaptation process by tumor cells to the host during repeated *in vivo* passaging cycles in nude rats most probably underlies the observed differences in engraftment rates between low generation and high generation spheroids in immunocompetent animals. Since T cell immunity is compromised in nude rats, we evaluated if the adaptation occurred to attenuate microglial infiltration into the xenograft tissue. In the CNS, microglia are primary sensors of pathology that react quickly to insult and possess a multitude of functions as antigen-presenting cells and producers of cytokines, bridging innate and adaptive immunity. We evaluated two independent markers known to be up-regulated on activated microglial cells: Allograft Inflammatory Factor-1 (AIF-1or Iba-1) and Cathepsin S. AIF-1 has been found to be expressed on monocytes in allograft rejection and on microglia in animal models of brain pathology [17, 18]. It has been described as a marker of glioma-infiltrating microglial cells [19]. Cathepsin S has similarly been found expressed on ED1+ activated macrophages and microglia associated with CNS injury [20]. Here, both markers identified the same population of cells based on cell shape and distribution. Cathepsin S staining showed a punctate pattern, consistent with its localization in cytoplasmic granules [21]. Immunopositive cells heavily infiltrated the tumor tissue in low generation P8 and P3 xenografts. The cells displayed predominantly intermediate and amoeboid microglial morphology, suggesting that they were in an activated state (Fig 7A). A proportion of immunopositive cells were perivascular macrophages, based on their rounded shape, lack of processes and location adjacent to tumor blood vessels. The numbers of AIF-1+ cells per high power field were significantly reduced in high generation xenografts (P8, median ± SD; 127.5 ± 73.8 vs. 57 ± 21.6; *P<*0.001; and in P3, 160.5 ± 23.6 vs. 65.5 ± 15.9, *P* = 0.002). Similarly, Cat S+ cells were dramatically reduced in high generation tumors, both in P8 (evaluated as area fractions per high power field: median ± SD; 0.049 ± 0.01 vs. 0.005 ± 0.01 SD; *P*<0.001) and in P3 xenografts (0.024 ± 0.013 vs. 0.007 ± 0.002; *P*<0.001).

To further evaluate the adaptation process, we performed human-specific gene expression arrays encompassing a broad panel of chemokines, cytokines and growth factors on corresponding low- and high generation xenografts of P3 and P8. Transcripts that were up- or down-regulated with at least a three-fold difference between low- and high generation xenografts are presented (Fig 7B). We found a strong attenuation of the levels of chemokines involved in leukocyte migration, such as CXCL10 and CXCL12 (and to a lesser extent, CXCL9) in high generation xenografts. A highly up-regulated transcript was CSF3 (or GM-CSF), described as an autocrine survival factor in gliomas [22]. Next, we performed additional quantitative RT-PCRs targeting selected human transcripts. Here, TGF-β2 showed consistent changes in both patient xenografts, with increased levels from corresponding low- to high generation tumors (Fold change ± SEM; 5.15 ± 0.65 in P8, 2.3 ± 0.1 in P3, Fig 7C), indicating the possible involvement of this potent immunomodulatory factor in the adaptation process.

## Discussion

Here, we have shown that human GBM cells passaged intracerebrally in nude rats are able to engraft in the brain of immunocompetent littermates, whereas primary biopsy spheroids are subject to chronic cellular graft rejection. Most likely, a concerted action of ED1+ monocytes, presumably acting as antigen presenting-cells and of primed T cells is necessary for rejection of human GBM xenografts in the rat CNS, since 1) primary GBM xenograft tissue consistently engrafts in T cell compromised nude rats (here, and [5]), but is invariably rejected in T cell sufficient immunocompetent littermates; 2) a tumor-to-host adaptation process occurs in nude rats that leads to a dramatic attenuation in the numbers of host microglia/macrophages. In the case of tolerance, leukocytes, therein CD4+ and CD8+ T cells fail to invade the tumor bed and are found only around tumor blood vessels. Although a high load of xeno-antigens were present, insufficient recruitment of CNS-resident APCs, together with limited access to the CNS for leukocytes in the circulation were most likely the cause of tolerance. We observed elevated proportions of Foxp3+ regulatory T cells within the CD4+ T cell compartment within tolerated xenografts. Sufficient levels of regulatory cells are crucial to enforce tolerance [23].

Although xenograft rejection in general is a function of the innate immune response [24, 25], it has become clear that CNS xenograft rejection is more dependent on MHC-restricted T cell immunity [26]. This fits well with the observed high intracerebral xenograft engraftment rate in nude rats, which have a well-developed innate immunological compartment, but lack mature functional T cells [16]. One may envision two ways of xenograft cell killing; an indirect way through presentation of foreign molecules by APCs to CD4+ T cells, which may express pro-inflammatory cytokines and promote killing by cytotoxic tissue macrophages or microglia. Second, direct recognition of foreign molecules presented on MHC class I receptor triggers CD8+ cytotoxic effector cells. Based on our data, both pathways have most likely contributed to tumor cell killing in this model.

Serum levels of IL-1α, IL-18 and TNF-α correlated with GBM xenograft rejection, reaching statistical significance on day 53 p.i. Several of these cytokines were similarly up-regulated in the brain of rats transplanted with fetal porcine neurons, indicating their importance in CNS xenograft rejection [26]. These cytokines are typically produced by activated macrophages, M1-like microglia and by reactive astrocytes and have a pro-inflammatory role, augmenting cytotoxic T cell responses [27]. Pro-inflammatory cytokines are also known to induce the expression of adhesion molecules such as ICAM-1 and VCAM-1 on the brain endothelium to promote entry of activated lymphocytes to the CNS [28].

To delineate the differences between low- and high generation xenografts that may have affected engraftment, we have validated candidate GBM-derived factors with roles in immunological responses. Significant increases in TGF-β2 transcript levels, and decreases in CXCL10 and CXCL12 were observed in corresponding low- and high generation tumors used in this study. TGF-β is an essential glioma-derived cytokine that specifically represses the proliferation of antigen-specific CD4+ T cells [29]. Since there is an interspecies cross-reactivity of TGF-β and their cognate receptors in mammals [30], human TGF-β2 may well have interfered with rat immunity. Tumor-derived CXCL10 and CXCL12 may have played a role in the massive infiltration of leukocytes to low generation xenografts. Conversely, down-regulation of these factors may have attenuated leukocyte recruitment and entry into the xenograft and the CNS. The CXCR3-CXCL10 axis has been demonstrated to underlie microglial migration in a rodent model of brain injury [31], and it also effects CD8+ T cell entry into the CNS [32]. CXCL12 (or SDF-1) is constitutively expressed in the CNS on blood vessels and astrocytes and is also found expressed in and around transplanted gliomas [4]. The tissue distribution of CXCL12 is important for the maintenance of BBB integrity; relocation from the basolateral- to the vessel luminal side of the BBB correlates with leukocyte entry into the CNS [33]. Increases in astrocyte-produced CXCL12 are seen in MS patients, where it correlates with the presence of leukocytes that express the ligand activated form of CXCR4 [33]. Differentially expressed interleukins were IL-5, IL-6 and IL-8. IL-6 is a known growth- and survival factor in glioblastomas. It seems to be expressed in tumors which present with increased perifocal edema, and is associated with neovasculature endothelia and inflammatory cells [34]. Ablation of IL-6 expression in mice genetically predisposed to glioblastomas prevents tumor formation, but not pre-neoplastic astrogliosis, indicating its role in neoplastic progression [35]. Concomitant up-regulation of IL-6 in both high generation xenografts may have contributed to the increased malignancy and survival potential of passaged tumors. Regarding its functions in immunology, IL-6 is required for T cell recruitment in peritoneal inflammation [36]. Whether GBM-produced human IL-6 affects the rat immune system is unknown. Host-derived IL-6 was analysed, but not detected in the sera of rats implanted with P3 xenografts, and it is well possible that human IL-6 contributed to T cell recruitment in our model. IL-8 was similarly up-regulated with passaging in both patient xenografts. As IL-6, IL-8 promotes angiogenesis and invasiveness of glioblastomas, and is an autocrine survival factor [37]. In immunology, IL-8 is known to be a specific activator of neutrophil granulocytes, but is chemotactic for a range of leukocytes as well. It inhibits inflammatory leukocyte adhesion to activated endothelial cells [38]. Human IL-8 does act on rodent cells [39], and its role in our system may well be attraction of leukocytes to the tumor, as observed in a model of renal allograft rejection, where the numbers of both granulocytes and ED1+ monocytes were affected [40]. To our knowledge, the expression of IL-5 in glioblastomas has not been described. Its functions in immunology mainly relate to eosinophil differentiation, and its role in our model is unknown.

Bone morphogenetic proteins are members of the TGF superfamily and are important regulators of neural differentiation. In our xenograft model, BMP-2 and 7 were differentially regulated. BMPs and their receptors are expressed by GBMs, and treatment of glioma cells with additional BMPs reduces proliferation and induces astroglial differentiation [41]. Of note, the amino acid sequences of human and rat BMP-2 are identical, thus biological activity of human BMP-2 in our system is expected. The expression of another TGF-β superfamily member, Myostatin was attenuated with passaging in both patient xenografts. Myostatin contributes to inflammatory conditions such as chronic kidney disease, and therapeutic blockage reduces immunopathology through the suppression of IL-6, IFN-γ and TNF-α [42]. Effects on the same cytokines in our model system may have contributed to immunosuppression.

In conclusion, we have shown that tolerance to human GBM xenografts in the xenogeneic brain is enabled by a combination of several factors, such as 1) inadequate infiltration of the xenograft tissue and the brain by leukocytes, 2) attenuated systemic levels of pro-inflammatory cytokines, 3) increases in the proportion of Foxp3+ regulatory T cells and reduction in the absolute numbers of Granzyme B+ effector cells and 4) an increase in the levels of tumor-produced immunosuppressive TGF-β2 and decreased levels of leukocyte-attracting chemokines. Our data suggest an adaptation of human GBM cells after serial passaging in nude rats to the xenogeneic environment. Such “adapted” tumor cells are able to engraft in the brain of immunocompetent rats without signs of an inflammatory response. The presented work also shows the feasibility of growing human GBMs in fully immunocompetent animals, which may be further evaluated in a therapeutic context.

## Supporting Information

S1 Fig Survival curves of immunocompetent rats implanted with glioblastoma biopsy spheroids. (A, B) Survival curves of immunocompetent rats implanted with spheroids derived from xenografts growing in immunocompetent (sph. from *rnu/-*) and nude rats (sph. from *rnu/rnu* spheroids).
(TIF)

S2 Fig Natural Killer cells and dendritic cells in the tumor bed and the brain of immunocompetent rats after transplantation of GBM xenografts. Panels show typical distribution of NKR-P1-positive and dendritic cell antigen-positive leukocyte subsets in the tumor and brain tissue of immunocompetent rats implanted with GBM xenografts. Shown are typical cases from immunocompetent rats with different engraftment outcomes. (A) An infiltrative GBM xenograft generated directly from patient tissue that elicited a strong immune response. (B) A diffusely growing, high generation GBM xenograft with significant immune response. (C) High generation GBM xenograft, tolerance. Scale bar: 100 μm. DCA-dendritic cell antigen (OX-62, related to CD103), NKR-P1 (CD161a).
(TIF)
