# Correction: Engraftment of Human Glioblastoma Cells in Immunocompetent Rats through Acquired Immunosuppression

**DOI:** 10.1371/journal.pone.0140303

**Published:** 2015-10-02

**Authors:** Peter C. Huszthy, Per Ø. Sakariassen, Heidi Espedal, Karl A. Brokstad, Rolf Bjerkvig, Hrvoje Miletic

There is an error in the fifth sentence of the Abstract. The correct sentence is: In contrast, rejection is associated with massive infiltration of the tumor bed by leukocytes, predominantly ED1+ microglia/macrophages, CD4+ T helper cells and CD8+ effector cells, and correlates with elevated serum levels of pro-inflammatory cytokines IL–1α, IL–18 and TNF-α.
