# Supplementary figures and images for: Engraftment of Human Glioblastoma Cells in Immunocompetent Rats through Acquired Immunosuppression
Source: PLoS One. 2015 Aug 20;10(8):e0136089. doi: 10.1371/journal.pone.0136089 (PMC4546393; doi:10.1371/journal.pone.0136089)

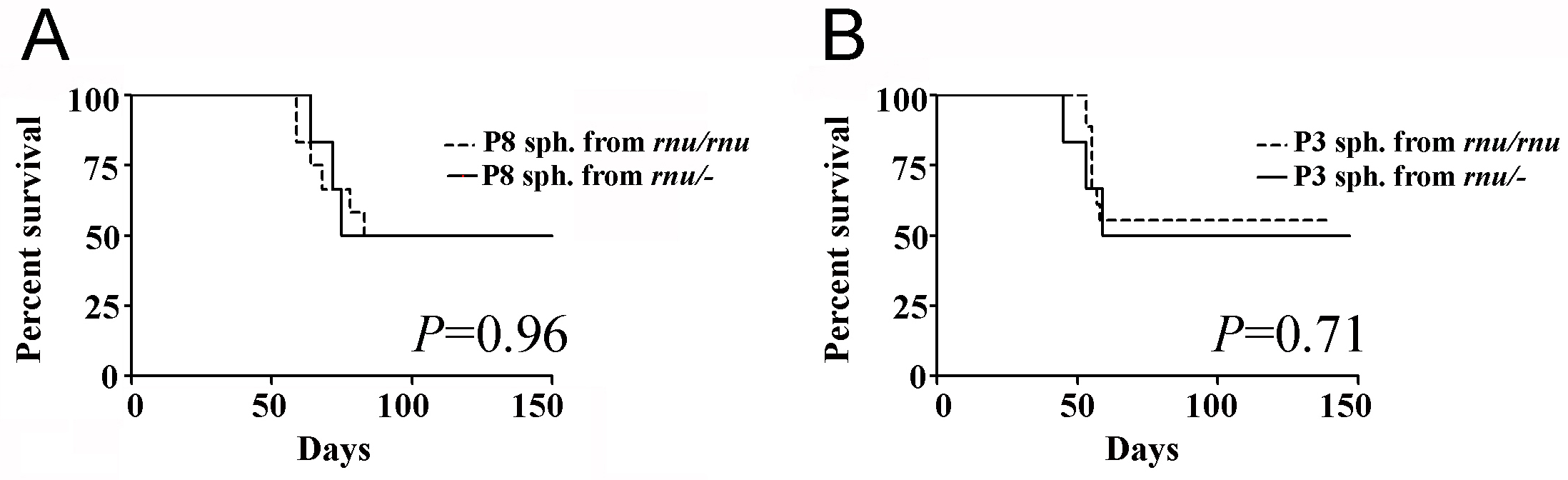

Supplement: S1 Fig — (A, B) Survival curves of immunocompetent rats implanted with spheroids derived from xenografts growing in immunocompetent (sph. from rnu/-) and nude rats (sph. from rnu/rnu spheroids). (TIF) [file pone.0136089.s001.tif]

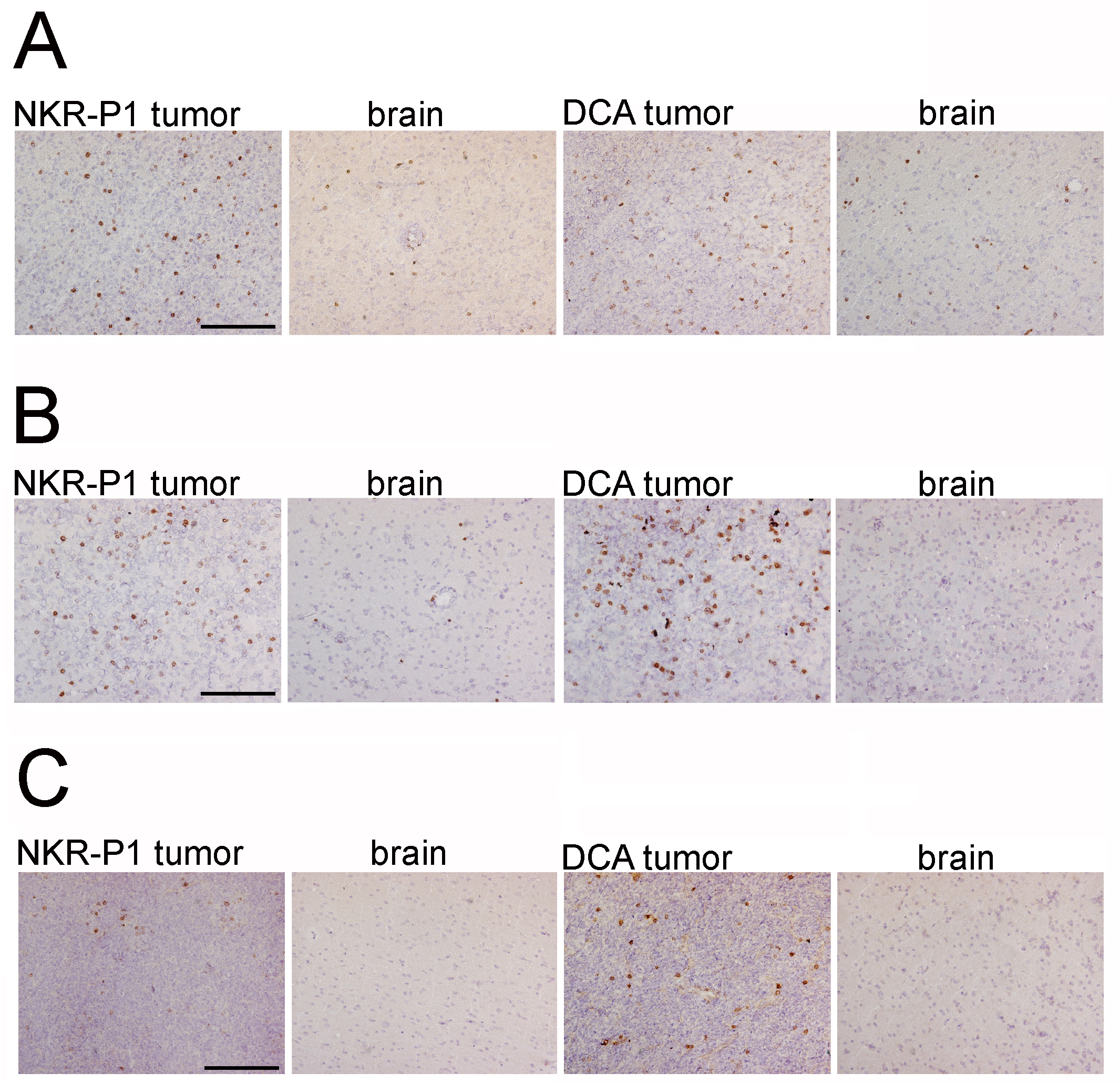

Supplement: S2 Fig — Panels show typical distribution of NKR-P1-positive and dendritic cell antigen-positive leukocyte subsets in the tumor and brain tissue of immunocompetent rats implanted with GBM xenografts. Shown are typical cases from immunocompetent rats with different engraftment outcomes. (A) An infiltrative GBM xenograft generated directly from patient tissue that elicited a strong immune response. (B) A diffusely growing, high generation GBM xenograft with significant immune response. (C) High generation GBM xenograft, tolerance. Scale bar: 100 μm. DCA-dendritic cell antigen (OX-62, related to CD103), NKR-P1 (CD161a). (TIF) [file pone.0136089.s002.tif]
